# Antibiogram for Haemodialysis Catheter-Related Bloodstream Infections

**DOI:** 10.1155/2014/629459

**Published:** 2014-01-22

**Authors:** Abdul Halim Abdul Gafor, Pau Cheong Ping, Anis Farahanum Zainal Abidin, Muhammad Zulhilmie Saruddin, Ng Kah Yan, Siti Qania'ah Adam, Ramliza Ramli, Anita Sulong, Petrick Periyasamy

**Affiliations:** ^1^Nephrology Unit, Department of Medicine, Universiti Kebangsaan Malaysia Medical Centre, Jalan Yaacob Latif, Bandar Tun Razak, Cheras, 56000 Kuala Lumpur, Malaysia; ^2^Universiti Kebangsaan Malaysia Medical Centre, Jalan Yaacob Latif, Bandar Tun Razak, Cheras, 56000 Kuala Lumpur, Malaysia

## Abstract

*Background*. Haemodialysis (HD) catheter-related bloodstream infections (CRBSIs) are a major complication of long-term catheter use in HD. This study identified the epidemiology of HD CRBSIs and to aid in the choice of empiric antibiotics therapy given to patients with HD CRBSIs. *Methods*. Patients with HD CRBSIs were identified. Their blood cultures were performed according to standard sterile technique. Specimens were sent to the microbiology lab for culture and sensitivity testing. Results were tabulated in antibiograms. *Results*. 18 patients with a median age of 61.0 years (IQR: 51.5–73.25) were confirmed to have HD CRBSIs based on our study criteria. Eight (44.4%) patients had gram-negative infections, 7 (38.9%) patients gram-positive infections, and 3 (16.7%) patients had polymicrobial infections. We noted that most of the gram-negative bacteria were sensitive to ceftazidime. Unfortunately, cloxacillin resistance was high among gram-positive organisms. *Coagulase-negative Staphylococcus* and *Bacillus* sp. were the most common gram-positive organisms and they were sensitive to vancomycin. *Conclusion*. Our study revealed the increased incidence of gram-negative organism in HD CRBSIs. Antibiogram is an important tool in deciding empirical antibiotics for HD CRBSIs. Tailoring your antibiotics accordingly to the antibiogram can increase the chance of successful treatment and prevent the emergence of bacterial resistance.

## 1. Introduction

Chronic kidney disease (CKD) is a major public health burden [1]. The contribution of CKD to the global burden of disease may be underestimated due to the lack of significant importance in certain kidney disease classifications and failure to realize the relationship of CKD with cardiovascular disease [1]. The prevalence of end-stage renal disease (ESRD) is increasing exponentially worldwide. USA, Japan, and Taiwan had the highest rate of prevalence of ESRD [2]. In Malaysia, dialysis is the main modality of renal replacement therapy. There were about 26,000 patients on dialysis in 2011 with the prevalence of 900 per million populations [3]. Haemodialysis (HD) accounts for about 89% of dialysis patients and most of them were accepted to centre HD [4]. Unfortunately, not all patients enter HD program with a native vascular access. Many patients still presented late and HD access catheter, either cuffed or noncuffed catheters, is needed to perform HD. The use of HD catheter had increased from 3% in 2002 to 8.1% in 2011 of all vascular accesses [5].

We cannot argue that HD catheter plays a very important role in the treatment of patients requiring HD. It is relatively easy to be inserted and can be used immediately in wide range of kidney failure patients. Unfortunately, HD catheter is not without problems. Beside thrombosis, infection is one of the most feared complications. Infection of the HD catheter was thought to cause an increase of >50% mortality in HD patients compared to patients on native fistulas and also cause significant morbidity in dialysis population [6]. The cause of HD catheter-related bloodstream infections (CRBSIs) is multifactorial ranging from patient's factors (i.e., comorbidities and hygiene) to catheter's factors (i.e., types of catheter and sites of insertion) [7].

Currently, the management of HD CRBSIs depends on the type of catheter involved and the severity of the infections. Antibiotics are the mainstay for the treatment of HD CRBSIs. Sometimes, the HD catheters would need to be replaced in complicated cases. It is important to initiate empirical antibiotic therapy before we receive the formal microbial reports. These empirical antibiotics should cover the gram-positive and gram-negative organisms. Each HD centre should maintain a database of all suspected and proven HD CRBSIs, with details on the causative organisms, their sensitivity to antibiotics, and the outcomes of therapeutic intervention. Moreover, each unit should know the epidemiology of its catheter-related infections [7].

Antibiogram is a list of antimicrobial susceptibilities of local bacteria isolated and produced by clinical microbiology laboratory. It has often been used by clinician to assess local susceptibility rates and select empirical therapy [8]. Each HD unit must have its own antibiogram to assist the nephrologist to choose empirical antibiotic for HD CRBSIs.

## 2. Methods

This study was approved by our hospital ethics and research committee (FF-006-2012). The study was conducted over 6 months in Universiti Kebangsaan Malaysia Medical Centre (UKMMC). It was a cross-sectional study which included ESRD patients with the diagnosis of HD CRBSIs. The diagnosis was made based on the clinical presentation of fever, chills and/or hypotension, and semiquantitative laboratory confirmation, when blood from the catheter demonstrates microbial growth at least 2 hours earlier than growth is detected in blood collected simultaneously from a peripheral vein [9, 10]. HD patients who presented with other source of infection were excluded from the study.

Consents were taken from the patients and demographic data were taken via interviews and reviews of patient's case files. Two sets of blood cultures were taken from each patient. One set of blood culture (anaerobic and aerobic) was taken from a peripheral vein and another set from the catheter. The peripheral blood culture was taken from a vein in the median cubital fossa or the flexor aspect of the forearm. A sterile zone was then demarcated by draping the area with a sterile sheet. The sterile zone was created by cleaning the area with 70% alcohol followed by 10% povidone-iodine in a circular motion starting from the centre and moving outwards, and the site was left to dry. Blood was taken from the catheter in a similar fashion. The catheter hub was then cleaned with 10% povidone-iodine and left to dry. An equal amount of blood was drawn for catheter and peripheral cultures. All operators wore plastic gowns, face masks, and sterile gloves to prevent contamination of the blood culture.

The blood cultures were then sent to our microbiology laboratory for culture and antibiotic sensitivity tests. All cultures isolated were tested using Clinical and Laboratory Standards Institute (CLSI) 2011 protocol.

## 3. Results

During the 6-month study period, 28 cases with suspected HD CRBSIs and positive blood cultures were identified. Nine cases were due to line colonization with no systemic infection and one case of bloodstream infection with an unknown primary source.

Eighteen patients with a median age of 61.0 years (IQR: 51.5–73.25) were confirmed to have HD CRBSIs based on our study criteria. Their baseline characteristic, isolated bacteria, and catheter outcome were tabulated in Table 1. Out of them, 8 (44.4%) patients had gram-negative infections, 7 (38.9%) patients had gram-positive infections and 3 (16.7%) patients had polymicrobial infections (Table 2).

**Figure 1:**
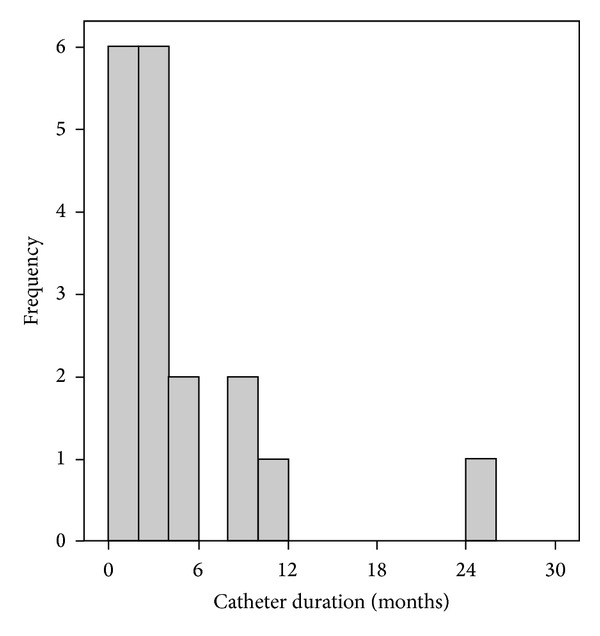
Haemodialysis catheter duration at the time of infection.

The median ESRD duration was 12 months (IQR: 6.50–39.0). Most of the patients (55.6%) were recently diagnosed with ESRD and started dialysis within the last 12 months.  Figure 1 shows the distribution of the catheter duration in the group of patients. The median catheter duration was 3 months (IQR: 1.00–5.00). The figure also showed the pattern of infection, where most cases (77.8%) happen within the first 6 months of catheter insertion.

The catheter was salvaged in 3 cases. All the cases where the line was salvaged were cuffed catheters.

Tables 3 and 4 were the antibiograms of gram-positive and gram-negative bacterial sensitivity testing. Each column represents the species of bacteria tested and the total number of bacteria isolated. The rows represent the different types of antibiotics tested for. Each bacteria-antibiotic combination was represented by the percentage of organisms sensitive to its antibiotic. Not all the bacteria isolated were tested for the same panel of antibiotics, as some bacteria were tested with antibiotics upon special request. The number of organisms tested was represented on the antibiogram as the number in parenthesis. Only vancomycin and linezolid were fully efficacious against gram-positive bacteria from the antibiogram. Cloxacillin was only effective against 40% of gram-positive bacteria. Cefepime was the most effective antibiotic with 100% sensitivity against gram-negative organisms tested. This was followed by amikacin, ceftazidime, and piperacillin-tazobactam which were effective towards 90% of gram-negative organisms tested.

## 4. Discussion

This study identified the epidemiology of HD CRBSIs in UKMMC. Most of our patients were diabetic and hypertensive and in concordance with the national HD patients' profiles [4]. A previous study by Jean et al. had shown that HD CRBSIs were more common in patients with diabetes mellitus [11]. This relationship is rather obvious as we know diabetic patients are more predisposed to develop infections due to their suppressed immunological state.

Qasaimeh et al. had shown that the causative organisms in HD CRBSIs were predominantly gram-positive cocci, followed by gram-negative bacilli, and polymicrobial infections [12]. In another study by Saad eon tunneled, cuffed, and permanent catheters showed that 45 out of 86 infections (52.3%) were caused by single gram-positive cocci. In that study, 23 infections (26.7%) were caused by single gram-negative rods only while 18 (20.9%) were polymicrobial [13]. According to a study by Schwab and Beathard, 84.5%, 33.3%, and 1.6% were caused by gram-positive cocci, gram-negative organisms, and acid-fast organisms, respectively. The most commonly reported isolate in these cases of catheter-related bacteraemia was *Staphylococcus aureus* [14]. Nasal carrier of *Staphylococcus aureus* is an important risk factor for HD CRBSIs, not only for gram-positive infections but also for gram-negatives and polymicrobial infections [11]. Thus, screening patients for carrier status is important and must be a routine procedure before accepting a patient into the HD program.

Our study was unusual as a high prevalence of gram-negative bacteraemia was found in HD patients. As compared to previous studies, our study showed an increasing trend of gram-negative bacteraemia. Alexandraki et al. investigated the five-year pattern of microbial isolated from HD patients with catheter infection, which showed a significant increase in the incidence of single gram-negative organisms and polymicrobial bacteraemias [15]. This trend was consistent with the trend of catheter infection in nondialysis patients [16, 17]. The high prevalence of gram-negative bacteria may be due to immunocompromised state of patients [16, 17], contaminated infusate [18], and misuse of antibiotics [19]. Thus, empirical antibiotic therapy for HD CRBSIs should include coverage for gram-negative organism and *Pseudomonas aeruginosa* infection in neutropenic patients [20].

Antibiogram is a list of laboratory testing for the sensitivity of an isolated bacterial strain to different antibiotics. In an era of bacterial resistance, a careful and correct selection of antibiotics is important to increase the chance of successful treatment and to reduce the rate of bacterial resistance. Antibiograms are often used by doctors to assess local susceptibility rates, to select empiric antibiotic therapy, and to monitor resistance trends within an institution [21]. Antibiograms are also used to compare susceptibility rates amongst institutions and bacterial resistance trends in the country [22]. Thus, antibiogram should be incorporated into the antibiotics assessment in each institution. Currently, antibiograms are only available in the larger hospitals with microbiology laboratory service. As the trend of bacterial resistance changes, antibiogram has to be reviewed regularly in timely manner.

Prior to this study, the empirical antibiotics for HD CRBSIs in our centre were intravenous cloxacillin and ceftazidime [23]. Based on this study, we noted that most of the gram-negative bacteria were sensitive to ceftazidime. Unfortunately, cloxacillin resistance was high among gram-positive organisms. We also realized that *Coagulase-negative Staphylococcus *and *bacillus *sp. were the most common gram-positive organisms and they were sensitive to vancomycin. Thus, following this study results, empirical antibiotics for HD CRBSIs in our centre were switched to intravenous vancomycin and ceftazidime.

## 5. Conclusion

Our study revealed the increased incidence of gram-negative organism in HD CRBSIs. We noted that antibiogram is an important tool in helping us to choose empirical antibiotics for HD CRBSIs. Tailoring your antibiotics accordingly to the antibiogram can increase the chance of successful treatment and prevent the emergence of bacterial resistance. Hence, we strongly urge each institution to have their own antibiogram in the management of HD CRBSIs. To provide a better representation of national infection patterns, data from multicenter studies could be incorporated. A yearly antibiogram will help to keep track of antibiotic resistance and also update the empirical antibiotic regime.

## Figures and Tables

**Table 1:** Baseline characteristics, bacterial isolated, and catheter outcome.

Patients' initials	Age (years)	Gender	Catheter type/site	Comorbidities	Bacteria isolated	Catheter outcome
AR	55	Male	Cuffed right IJV	DM/HPT	*Bacillus *sp.	Removed
AA	61	Male	Noncuffed right IJV	HPT	*Stenotrophomonas* sp. *Citrobacter* sp.	Removed
FFY	85	Female	Noncuffed right IJV	DM/HPT	MSSA *Bacillus *sp.	Removed
FSW	29	Female	Cuffed right IJV	DM/HPT	*Pseudomonas* sp.	Salvaged
HMJ	83	Male	Cuffed left IJV	DM/HPT	*Flavobacterium* sp.	Removed
LHS	61	Male	Noncuffed right IJV	HPT	CONS	Removed
MY	32	Male	Noncuffed right FV	HPT	*Morganella* sp.	Removed
MAMA	60	Male	Cuffed right IJV	DM/HPT	*Pseudomonas* sp.	Removed
MAK	73	Male	Cuffed right IJV	DM/HPT	*Stenotrophomonas* sp.	Salvaged
MNO	52	Male	Noncuffed right IJV	DM/HPT	CONS	Removed
OKK	61	Male	Noncuffed right IJV	DM/HPT	MRSA	Removed
OTS	75	Male	Cuffed left IJV	DM/HPT	*Serratia* sp.	Salvaged
PM	62	Male	Cuffed right IJV	DM/HPT	*Enterobacter* sp.	Removed
PI	68	Female	Noncuffed right IJV	DM	*Enterobacter* sp.	Removed
SA	40	Female	Cuffed left IJV	HPT	*Bacillus *sp. *Stenotrophomonas* sp.	Removed
WKY	74	Male	Cuffed right IJV	HPT	MSSA	Removed
YI	72	Male	Noncuffed right IJV	DM/HPT	CONS	Removed
YSP	50	Female	Cuffed right IJV	HPT	*Enterobacter* sp.	Removed

IJV: internal jugular vein; FV: femoral vein; DM: diabetes mellitus; HPT: hypertension; methicillin sensitive *Staphylococcus aureus *(MSSA); methicillin resistant *Staphylococcus aureus* (MRSA); *coagulase-negative Staphylococcus* (CONS).

**Table 2:** Bacterial isolates from 18 blood cultures.

	Count (%)
Gram-positive organisms	
*Coagulase-negative Staphylococcus* (CONS)	3 (14.3)
*Bacillus *sp.	3 (14.3)
Methicillin sensitive *Staphylococcus aureus *(MSSA)	2 (9.52)
Methicillin resistant *Staphylococcus aureus* (MRSA)	1 (4.76)
*Enterococcus* sp.	1 (4.76)
Total	**10 (47.6) **
Gram-negative organisms	
*Stenotrophomonas* sp.	3 (14.3)
*Pseudomonas* sp.	2 (9.52)
*Enterobacter* sp.	2 (9.52)
*Citrobacter* sp.	1 (4.76)
*Flavobacterium* sp.	1 (4.76)
*Morganella* sp.	1 (4.76)
*Serratia* sp.	1 (4.76)
Total	**11 (52.4) **

Total for all organisms	**21 (100) **

**Table 3:** Antibiogram for gram-positive bacteria.

Bacteria	*Bacillus *sp.	CONS	MSSA	MRSA	*Enterococcus * sp.
Number of isolates	3	3	2	1	1
	Percentage (%)				
Amikacin	100 (3)				
Ciprofloxacin		33.3 (3)	50 (2)	0 (1)	
Clindamycin		33.3 (3)	50 (2)	0 (1)	
Doxycycline		66.7 (3)	50 (2)	100 (1)	
Erythromycin		33.3 (3)	50 (2)	0 (1)	
Fusidic acid		33 (3)	50 (2)	100 (1)	
Gentamicin		100 (3)	100 (2)	0 (1)	0 (1)
Imipenem					
Linezolid		100 (3)	100 (2)	100 (1)	100 (1)
Mupirocin		66.7 (3)	100 (2)	0 (1)	
Netilmicin	100 (3)				
Cloxacillin		0 (2)	100 (2)	0 (1)	
Penicillin G		0 (3)	50 (2)	0 (1)	100 (1)
Piperacillin-tazobactam	33.3 (3)		100 (1)		
Rifampicin		100 (3)	100 (2)	0 (1)	
Teicoplanin	100 (1)	100 (3)	50 (2)	100 (1)	100 (1)
Tetracycline	100 (2)				
Trimethoprim-sulfamethoxazole		33.3 (3)	100 (2)	0 (1)	
Vancomycin	100 (3)	100 (1)	100 (1)		100 (1)

*Number in parenthesis is the number of isolates tested for that particular bacteria-antibiotic combination.

**Table 4:** Antibiogram for gram-negative bacteria.

Bacteria	*Citrobacter freundii *	*Enterobacter cloacae *	*Flavobacterium* sp.	*Morganella morganii *	*Pseudomonas * sp.	*Serratia* sp.	*Stenotrophomonas maltophilia *
Number of isolates	1	2	1	1	2	1	3
	Percentage (%)						
Amikacin	100 (1)	100 (2)		100 (1)	50 (2)	100 (1)	100 (3)
Augmentin	0 (1)	0 (2)		0 (1)	0 (1)	0 (1)	0 (1)
Cefepime	100 (1)	100 (2)		100 (1)	100 (2)	100 (1)	100 (3)
Cefotaxime	100 (1)	100 (2)		0 (1)	0 (1)	100 (1)	0 (1)
Ceftazidime	100 (1)	100 (2)		100 (1)	50 (2)	100 (1)	100 (3)
Ciprofloxacin	100 (1)	100 (2)	100 (1)	0 (1)	100 (2)	100 (1)	66.7 (3)
Doxycycline							
Erythromycin			0 (1)				
Gentamicin	100 (1)	100 (2)		100 (1)	50 (2)	100 (1)	66.7 (3)
Imipenem	100 (1)	100 (2)	0 (1)	0 (1)	50 (2)	100 (1)	0 (3)
Meropenem	100 (1)	100 (2)		100 (1)	100 (2)	100 (1)	0 (3)
Piperacillin-tazobactam	100 (1)	100 (2)		100 (1)	50 (2)	100 (1)	100 (3)
Polymyxin B					0		100 (3)
Rifampicin			100 (1)				
Trimethoprim-sulfamethoxazole			0 (1)	0 (1)	100 (2)		100 (3)
Vancomycin			100 (1)				

*Number in parenthesis is the number of isolates tested for that particular bacteria-antibiotic combination.
